# QuickStats: Age-Adjusted Drug Overdose Death* Rates,^†^ by State — United States, 2018^§^

**DOI:** 10.15585/mmwr.mm6915a5

**Published:** 2020-04-17

**Figure:**
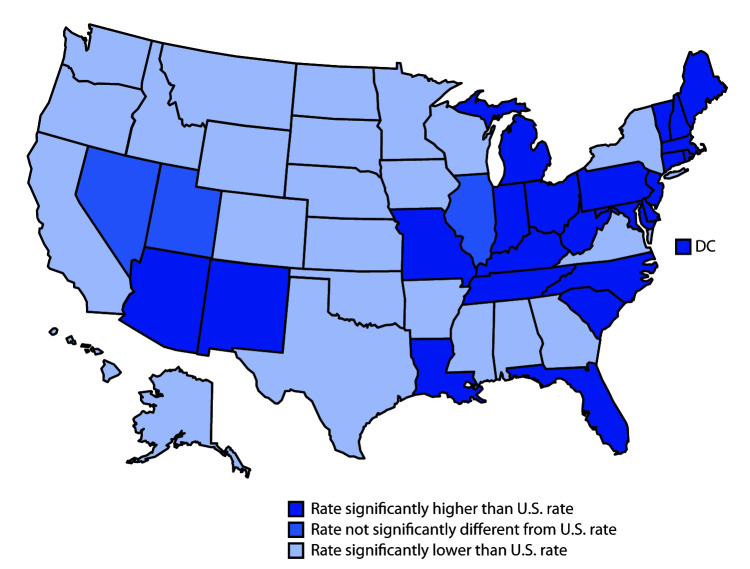
In 2018, 23 states and DC had drug overdose death rates that were higher than the national rate of 20.7 per 100,000. Except for Arizona and New Mexico, states with higher rates were in the eastern part of the country, including the two states with the highest rates: West Virginia (51.5) and Delaware (43.8). Twenty-four states had rates that were lower than the national rate; the states with the lowest rates were Nebraska (7.4) and South Dakota (6.9). Three states (Illinois, Nevada, and Utah) had rates that were not statistically different from the national rate.

**Source:** National Center for Health Statistics, National Vital Statistics System, Mortality Data. https://www.cdc.gov/nchs/deaths.htm.

For more information on this topic, CDC recommends the following link: https://www.cdc.gov/drugoverdose/.

